# The Strange Case of Dr. Bruno*Editorial by Professor Hughes, the veteran editor of the *Alienist and Neurologist*.

**Published:** 1914-02

**Authors:** F. E. Daniel


THE STRANGE CASE OF DR. BRUNO.*—By F. E. Daniel, M. D. Dr. Daniel is a master of scientific fact, and knows how to make it entertaining in the reading. His fact is as interestingly portrayed as his fiction.

The strange case of Dr. Bruno might have been written by Robert Louis Stevenson, but the portrayal of the story could be no better than if written by the author of Dr. Jekyll and Mr. Hyde, nor any better if a Hawthorne had written it instead of Archibald Malmaison.

This story weaves in facts of psychopathological science, interesting to the medical profession and all students of psychology and psychological mystery as well as to the intelligence-seeking general reader.

Changes of character, double and alternate personality as described by Wigan, Mitchel, James and the present reviewer, as well as an interesting explanation with neurone anatomical plate (schematic) of Reuchard's conception of the phenomenon and mechanism of sleep.

The author's skillful blending of psychologic truth with fiction is here most skillfully done, and equal to the best writers known to the reviewer in connection with similar portrayals, entertaining at once the science-searching mind for fact and imagination reveling mind of the fiction lover. The case related from the present reviewer is true to the observed fact.

The scenes and history of this "strange case of Dr. Bruno" are largely in the Southern States of the American Union, and incidentally illustrate the power of music and, especially in this case, the potent psychotherapy of the violin in recalling to normal con-

*Editorial by Professor Hughes, the veteran editor of the *Alienist and Neurologist*.

sciousness of this singular amnesic victim medicinally self-induced and time-limited hypnosis.

Dr. Daniel, the talented author of this remarkable story, is the well known editor of the TEXAS MEDICAL JOURNAL, generally called the "Red Back."

The publisher is the Von Boeckmann-Jones Co., Austin, Texas. Price, $1.00.—*Alienist and Neurologist.*

---

If a cursory examination of a radiograph shows no fracture, in a case where clinical signs indicate its presence, look carefully within the bone shadow for the evidence of a fissuring or subperiosteal break.—*American Journal of Surgery.*

